# A Case of Hypoglycemia Associated With Anorexia Nervosa Revealing Isolated Adrenocorticotropic Hormone (ACTH) Deficiency

**DOI:** 10.7759/cureus.79383

**Published:** 2025-02-20

**Authors:** Shunsuke Hayashi, Taishi Ando, Kohei Nakano

**Affiliations:** 1 Department of Diabetes and Endocrinology, Japan Community Health Care Organization (JCHO) Tokuyama Central Hospital, Shunan, JPN; 2 Department of Hematology, Japan Community Health Care Organization (JCHO) Tokuyama Central Hospital, Shunan, JPN; 3 Department of Internal Medicine, Yamaguchi University Hospital, Ube, JPN; 4 Department of Diabetes, Endocrinology and Metabolism, Yamaguchi General Medical Center, Hofu, JPN

**Keywords:** anorexia nervosa (an), hypoglycemia, hypopituitarism, isolated acth deficiency, nutritional deficiency

## Abstract

Anorexia nervosa (AN) typically affects young women and leads to severe emaciation, while isolated adrenocorticotropic hormone (ACTH) deficiency (IAD) usually occurs in middle-aged and older adults, causing secondary adrenal insufficiency, appetite loss, and weight loss. Although differentiating between these conditions can be challenging, their coexistence is rare. We encountered a patient with AN hospitalized for impaired consciousness due to hypoglycemia, who was later diagnosed with IAD. It is important to consider IAD as a differential diagnosis when hypoglycemia occurs in patients with AN.

## Introduction

The anterior pituitary gland secretes six types of hormones: adrenocorticotropic hormone (ACTH), thyroid-stimulating hormone (TSH), growth hormone (GH), luteinizing hormone (LH), follicle-stimulating hormone (FSH), and prolactin (PRL). Among these, ACTH stimulates the secretion of cortisol, an adrenal cortex hormone. Adrenal cortex hormones are stress-resistant and play roles in metabolism and anti-inflammatory processes. Deficiency of cortisol causes adrenal insufficiency, leading to decreased appetite, weight loss, hypoglycemia, hypotension, and hyponatremia. If the deficiency is severe, it can cause impaired consciousness and shock, and delayed cortisol replacement can result in a fatal outcome. Adrenal insufficiency is broadly classified into primary adrenal insufficiency (known as Addison’s disease), which results from decreased adrenal function, and secondary adrenal insufficiency, in which cortisol deficiency arises from reduced ACTH secretion due to decreased function of the anterior pituitary (or hypothalamus).

Isolated ACTH deficiency (IAD) is a condition in which only ACTH secretion from the anterior pituitary is impaired due to trauma or an autoimmune mechanism, leading to secondary adrenal insufficiency [1]. Surveys in regions of Japan with a high number of elderly people report a prevalence of three to seven people per 100,000 population [2].

Anorexia nervosa (AN) is characterized by significant weight loss and severe emaciation due to voluntary dietary restrictions, which can sometimes be fatal due to nutritional deficiencies. AN is common in teenagers, with a prevalence of 0.3% in young women [3]. AN is a psychiatric disorder; AN patients have hormonal abnormalities due to starvation, but hormone deficiency is not the cause of AN.

Reports of the coexistence of AN and IAD are rare. Patients with AN typically present predominantly with symptoms such as general fatigue and loss of appetite. Although clinical differentiation is often possible, symptoms like weight loss and hypoglycemia are common in both conditions, making differentiation occasionally challenging. There have been reports where AN was initially suspected, but a thorough examination led to a diagnosis of adrenal insufficiency [4], but reports of concurrent AN and IAD are nearly nonexistent. In this case, we encountered a patient who was hospitalized with impaired consciousness due to hypoglycemia during the long-term course of AN and was diagnosed with concomitant IAD after a thorough examination. Because reports of both diseases coexisting are rare, hypoglycemia associated with AN tends to be attributed to malnutrition. In patients with IAD, however, delayed cortisol replacement can be lethal. Therefore, accurate diagnosis is important.

We have obtained consent from the patient and her family to present the results of this study. A summary of this case was presented at the 62nd Annual Meeting of the Chugoku-Shikoku Branch of the Japan Diabetes Society.

## Case presentation

The patient is a 62-year-old woman. Four years before admission, she had lost weight through dieting in her youth and had been eating less for over 10 years due to stress at work. She had decreased kidney function and hypokalemia and had been attending the urology outpatient clinic at our hospital. A blood test showed ACTH at 36 pg/mL (7.2-63.3), which was not decreased, ruling out IAD. She was extremely underweight, with a height of 145.9 cm, a weight of 26.8 kg, and a BMI of 12.5. There were abnormal eating behaviors, such as overeating and vomiting, and the weight loss led to a diagnosis of AN. Although a psychiatric consultation was recommended, she was refused. With her husband's assistance, she managed her daily life. 

She had been experiencing an exacerbation of appetite loss almost a year before admission, although hypotension was unclear.

**Figure 1:**
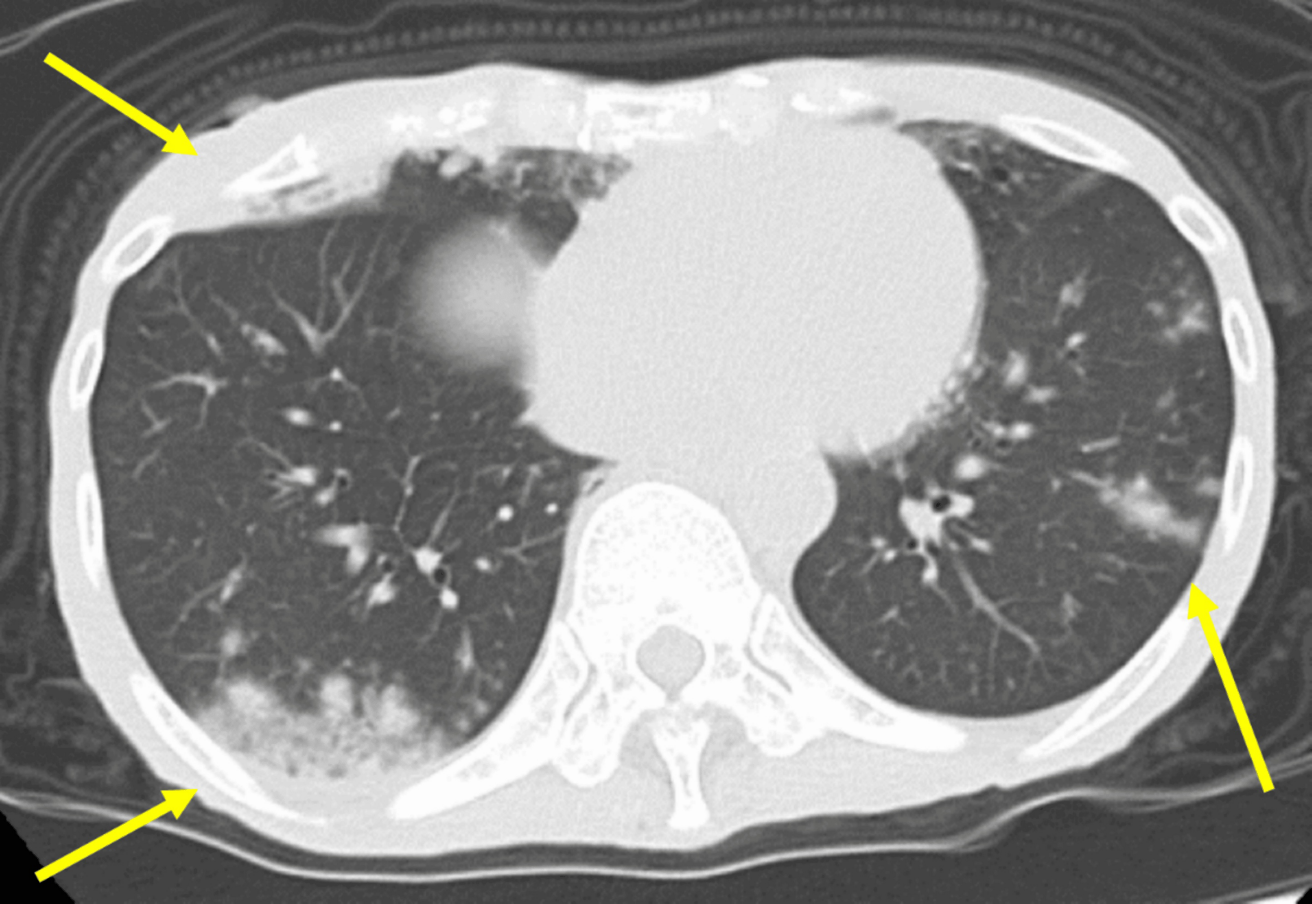
Chest CT on admission shows pneumonia.

**Figure 2:**
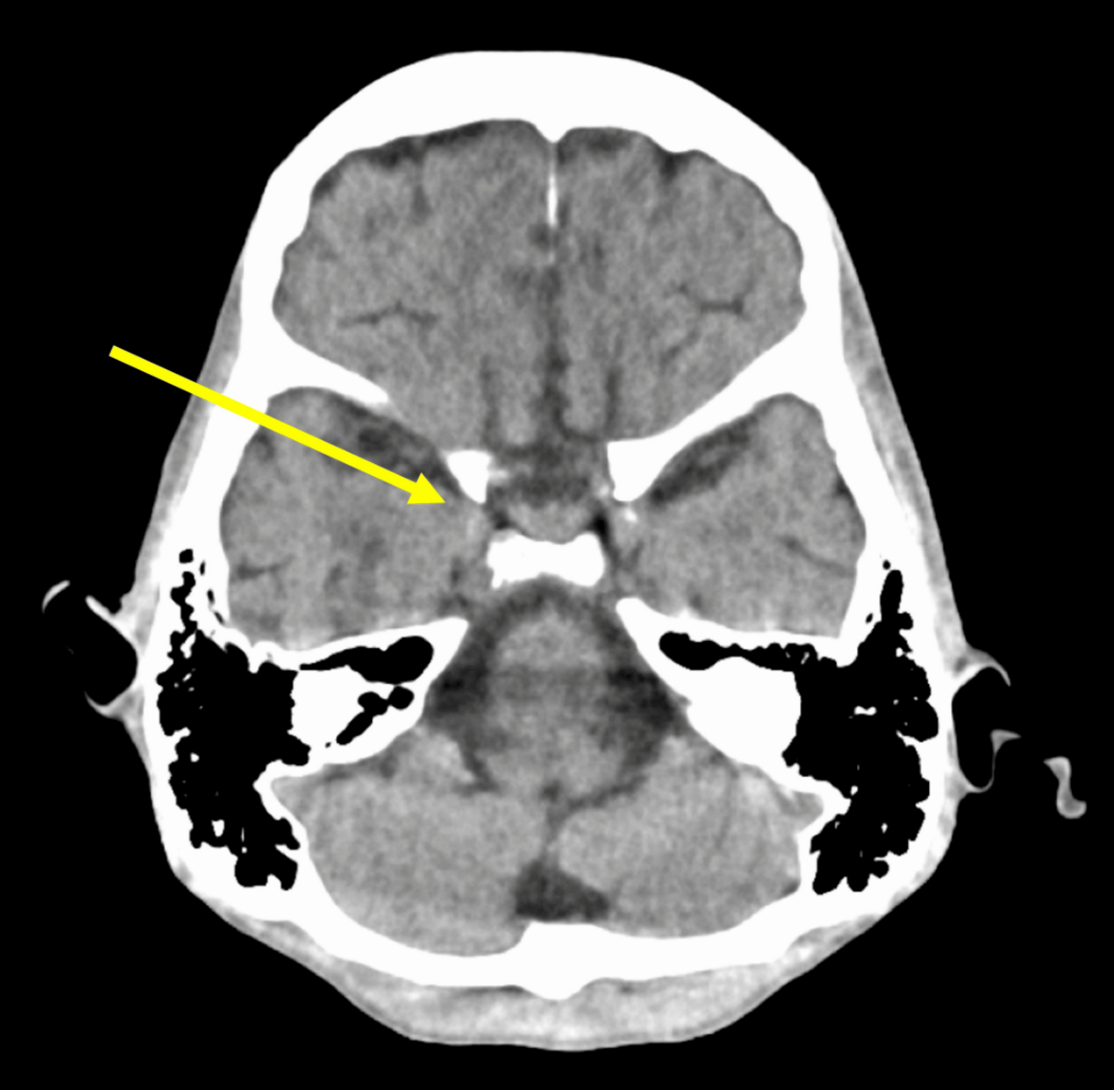
CT of the head shows no significant findings, including the pituitary gland.

Four years later, in the spring of the early 2020s, she was brought to our hospital by emergency transport due to impaired consciousness and was found to have a blood glucose level of 37 mg/dL (73-109), which improved with intravenous glucose. Serum sodium was normal at 143 mg/dL. She was severely emaciated, with thin arms and legs. Her weight was 28.6 kg, unchanged from four years prior. Based on her history, we determined that she has AN. A chest CT showed signs of pneumonia (Figure 1), and a head CT showed no significant abnormalities (Figure 2). According to the patient, her anorexia had been gradually worsening for almost a year. It was determined that her hypoglycemia was triggered by an infection against the background of malnutrition due to AN, and she was admitted the same day.

After hospitalization, a soft meal of 1600 kcal was provided. Her food intake fluctuated greatly, but she generally consumed about half of her meals, with a daily calorie intake of about 800 kcal. She frequently experienced hypoglycemia, with blood glucose levels of 50-60 mg/dL in the morning and evening when hungry, necessitating glucose administration. Blood tests during hypoglycemia showed that her blood C-peptide was suppressed below sensitivity, ruling out excessive insulin secretion from insulinoma, etc. (Table 1).

**Table 1:** Blood test at admission ACTH: adrenocorticotropic hormone

Blood test	Case	Reference
White blood cell (/µL)	9720	3300-8600
Red blood cell (10^4^/µL)	433	386-492
Hemoglobin (g/dL)	12.1	11.6-14.8
Hematocrit (%)	37.8	35.1-44.4
Platelet (×10^4^/µL)	26.7	15.8-34.8
Sodium (mmol/L)	143	138-145
Potassium (mmol/L)	4.42	3.6-4.8
Chloride (mmol/L)	107	101-108
Calcium (mmol/L)	2.3	2.2-2.5
Phosphorus (mmol/L)	1.5	0.87-1.5
Urea nitrogen (mg/dL)	23.1	8-20
Creatinine (mg/dL)	1.29	0.46-0.79
C-reactive protein (mg/dL)	8.48	0-0.14
ACTH (pg/mL)	<1.5	7.2-63.3
Cortisol (µg/dL)	2.1	4.5-21.1
Cortisol in urine (µg/day)	1.0	4.3-176
Blood glucose (mg/dL)	37	73-109
Insulin (µU/mL)	<0.4	0-18.7
C-peptide (ng/mL)	0.1	0.8-2.5

**Figure 3:**
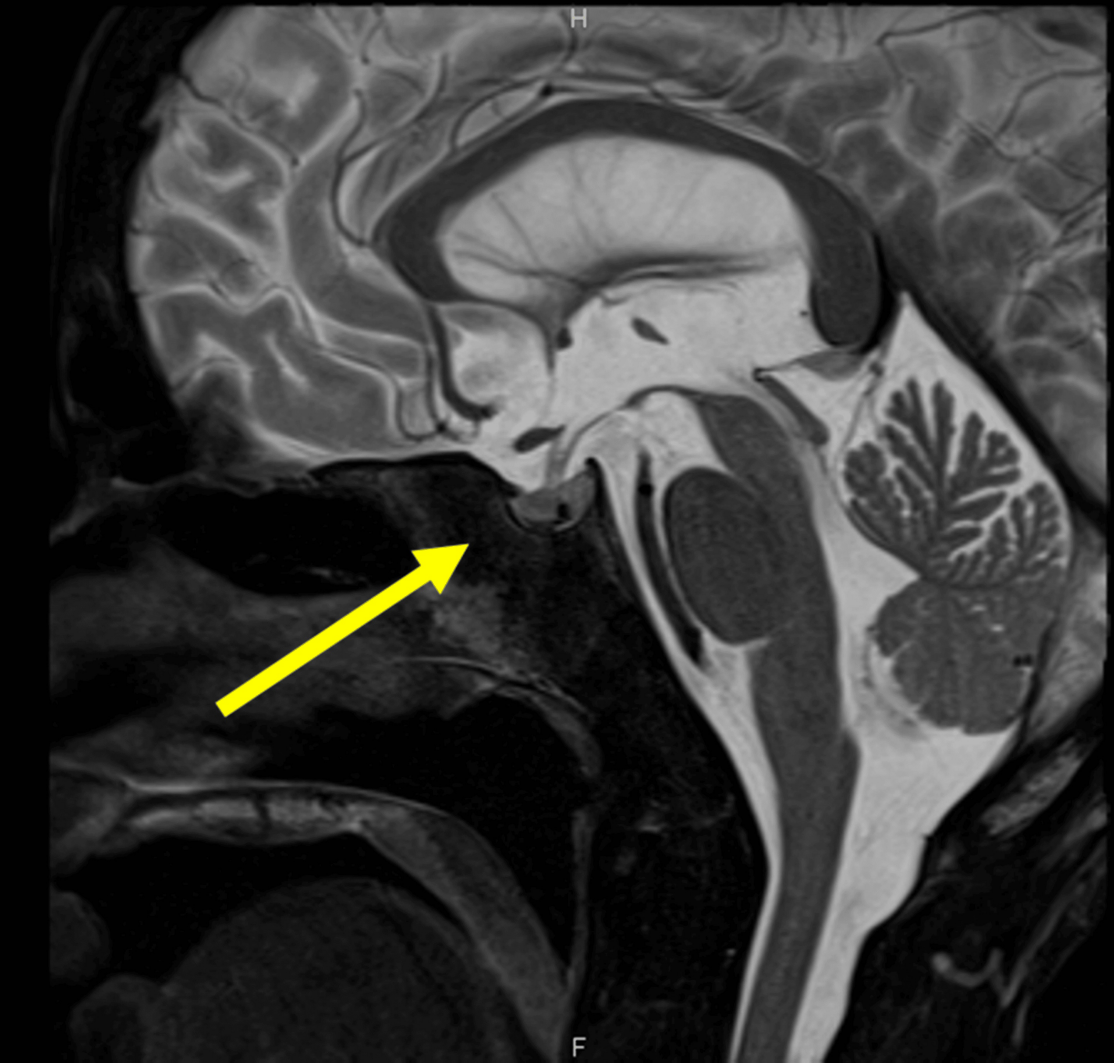
MRI of the head shows no significant findings in the pituitary gland.

Anti-insulin antibodies were negative, ruling out insulin autoimmune syndrome. The endocrinological evaluation showed decreased baseline ACTH and cortisol levels. The 24-hour urinary cortisol was low. TSH, LH, and FSH responded to corticotropin-releasing hormone (CRH), thyrotropin-releasing hormone (TRH), and luteinizing hormone-releasing hormone (LH-RH) stimulation tests. However, ACTH and cortisol did not (Table 2). She developed COVID-19 during hospitalization, so treatment was provided, and hydrocortisone administration was started. Hypoglycemia was still observed before breakfast, but the severity improved compared to before starting hydrocortisone. Two weeks after hospitalization, her food intake did not significantly change, and there was no weight gain, but she was deemed capable of daily life and was discharged after 17 days of hospitalization with hydrocortisone 15 mg/day. A plain head MRI performed one month later showed no significant abnormalities in the pituitary gland and other areas (Figure 3). Based on the above course and findings, the diagnosis was made that AN was complicated by IAD, which caused hypoglycemia.

**Table 2:** Result of CRH/TRH/LH-RH test (CRH 100 µg, TRH 200 µg, intravenous bolus) ACTH: adrenocorticotropic hormone, CRH: corticotropin-releasing hormone, FSH: follicle-stimulating hormone, LH: luteinizing hormone, LH-RH: luteinizing hormone-releasing hormone, PRL: prolactin, TRH: thyrotropin-releasing hormone, TSH: thyroid-stimulating hormone

	0 minutes	30 minutes	60 minutes	90 minutes
ACTH (pg/mL)	1.5	1.5	1.5	-
Cortisol (µg/dL)	0.2	-	0.2	0.2
TSH (µIU/mL)	22	100	100	-
PRL (ng/mL)	19	106	81	-
LH (U/L)	1.8	28	29	-
FSH (U/L)	5.3	-	23	25

## Discussion

This case highlighted the possibility of co-occurrence of AN and IAD. It also became clear that when a patient with AN experiences hypoglycemia, it is important to consider IAD as a differential diagnosis.

Although rare, AN and IAD can coexist. AN is more common in women and causes various complications due to severe nutritional disorders [5]. Resistance to food intake, noticeable low body weight, and emaciation are prominent features. While it can sometimes be easily diagnosed based on its characteristic clinical findings, differential diagnosis with other diseases can be problematic. Differential diagnoses of AN include diseases that cause loss of appetite and weight loss, such as cancer, inflammatory bowel disease, infections, other mental disorders like schizophrenia, and brain tumors [6]. Brain tumors near the hypothalamus can present symptoms similar to AN, complicating the differentiation from AN [7]. Adrenal insufficiency presents with symptoms like generalized fatigue, loss of appetite, and weight loss, which are common with AN, making differentiation challenging. There have been multiple reports where AN was initially suspected but later diagnosed as Addison's disease instead [8-10]. The papers above were reports about patients diagnosed with AN but later diagnosed with another disease. Differentiating IAD from AN is sometimes problematic. Reports of both coexisting are rare, but they can occur together.

When hypoglycemia occurs in AN patients, it is necessary to differentiate IAD. Hypoglycemia requires differentiation from a wide range of diseases. Symptomatic hypoglycemia can be caused by those related to diabetes treatment, reactive hypoglycemia, dumping syndrome, insulinoma, insulin autoimmune syndrome, malnutrition, or adrenal insufficiency. It is also known that hypoglycemia can occur in the early stages of sepsis [11]. AN can cause hypoglycemic coma due to nutritional disorders, which can sometimes be fatal [12]. Hypopituitarism can also present with hypoglycemia as the initial symptom due to secondary adrenal insufficiency [13]. Diagnosing the cause of hypoglycemia and providing appropriate treatment according to the disease can improve the patient's prognosis. IAD is one of the differential diseases in hypoglycemia in AN patients.

This case is considered to have had AN for a long time, although the time of onset is unknown. The patient had dieted in her youth and had a decreased food intake due to work-related stress for over 10 years. About four years ago, based on a low BMI and clinical course, the patient was diagnosed with AN. Hypokalemia, hypophosphatemia, and renal impairment were observed, and it was determined to be Pseudo-Bartter syndrome associated with AN. Bartter syndrome is a congenital disorder that causes salt loss and hypokalemia. Patients with AN may develop hypokalemia and metabolic alkalosis due to vomiting or diuretic and laxative abuse, a condition known as Pseudo-Bartter syndrome. Blood tests at this time showed high ACTH levels, ruling out pituitary insufficiency. 

The patient was diagnosed with IAD based on hormone levels and load testing. In primary adrenal insufficiency, serum cortisol levels are low, and ACTH levels are high due to negative feedback. In secondary adrenal insufficiency, ACTH levels and cortisol levels are low due to pituitary abnormalities. IAD is a disorder in which only ACTH production is impaired among pituitary hormones. Patients with AN have dysfunction in the hypothalamic-pituitary-adrenal axis, with high basal levels of blood ACTH and cortisol but weak responsiveness [14,15]. This finding is considered useful for differentiating between AN and IAD. In this case, the basal levels of ACTH and cortisol were low, and the 24-hour urinary-free cortisol level was low. In a hormone load test, pituitary hormones respond to stimulation, whereas only ACTH does not respond to CRH stimulation. TSH, LH, and FSH were maintained. Insulin-like growth factor-1 (IGF-1) is low in AN patients due to malnutrition [16]. Serum sodium levels were normal. In IAD, secondary adrenal insufficiency, hyponatremia, and hypotension may be less prominent than in primary adrenal insufficiency if mineralocorticoids are maintained [1]. The absence of abnormal findings in the pituitary gland on MRI and the improvement of symptoms after hydrocortisone administration support this diagnosis.

Generally, when hydrocortisone is started in patients with IAD, appetite loss typically improves, and food intake increases. Because COVID-19 developed at the same time hydrocortisone was started, it is not known whether the fatigue and other symptoms associated with adrenal insufficiency improved immediately after hydrocortisone was started. The patient visited the outpatient clinic one month later and reported feeling better and having an increased appetite. Her weight remained low, and it is unclear whether her food intake at home increased. A year later, she reported feeling better but not gaining weight. While symptoms due to adrenal insufficiency improved with hydrocortisone administration, AN did not improve. It was considered that due to long-term AN and originally low food intake, there were no changes in food intake or weight. Continued hydrocortisone administration and careful follow-up are important to maintain the patient's health in the future. Psychological support should also be considered.

We have previously described some case reports of patients who were initially thought to have AN but were later diagnosed with Addison's disease. The differences between these cases from our case are as follows: (1) they are young or teenagers, (2) the diagnosis is Addison's disease rather than IAD, and (3) they recover after treatment is started, so they are not thought to have AN at the same time. The incidence of AN is high in this age group, and Addison's disease can also develop in the teenage years as an autoimmune disease.

AN and IAD are not uncommon diseases. One reason for the rarity of their concurrence is the difference in the age of onset. The lifetime prevalence of AN in women is said to be up to 4%, making it not a rare disease [17]. AN generally occurs more frequently in adolescents and young women. IAD is also not extremely rare in Japan. While the onset of IAD spans a wide range of ages, excluding congenital cases, it often occurs in middle age or later. The aforementioned report from Japan states that the age of onset ranges from 49 to 77 years [2].

Recently, the presence of elderly AN patients has been gaining attention [18,19]. AN can develop in youth and persist into old age, or it can onset at an older age. In such cases, the disease can last for several decades. Older AN patients may be more susceptible to a variety of other diseases as they age. In this case, the patient developed IAD during a long time course of AN.

## Conclusions

We reported a case where a patient experienced symptomatic hypoglycemia during the long-term course of AN, and a subsequent thorough examination diagnosed the patient with IAD. AN patients can develop a variety of diseases as they age. This case revealed that AN and IAD can coexist. Furthermore, it highlighted the importance of considering IAD as a differential diagnosis when a patient with a history of AN develops hypoglycemia, not just attributing it to nutritional disorders. Early detection and appropriate management of IAD are crucial for preventing complications in such complex clinical situations and optimizing patient outcomes.
